# DuAL-Net: A Dual-Network Approach for Alzheimer’s Disease Risk Prediction Using *APOE*-Centered Regional Whole-Genome Sequencing Data

**DOI:** 10.34133/csbj.0010

**Published:** 2026-03-11

**Authors:** Eun Hye Lee, Taeho Jo

**Affiliations:** Indiana Alzheimer Disease Research Center and Center for Neuroimaging, Department of Radiology and Imaging Sciences, Indiana University School of Medicine, Indianapolis, IN 46202, USA.

## Abstract

Alzheimer’s disease prediction using genomic data remains challenging due to the high dimensionality of whole-genome sequencing data and the complex relationships between genetic variants. We developed DuAL-Net (Dual Approach Local-global Network), a hybrid framework that integrates local genomic window analysis with global annotation-based modeling to prioritize disease-associated single-nucleotide polymorphisms (SNPs). As a proof of concept, we applied DuAL-Net to 14,094 SNPs within the *APOE* ±50-kb region from 1,050 individuals in the Alzheimer’s Disease Neuroimaging Initiative and Alzheimer’s Disease Sequencing Project (ADSP) cohorts. Using nested 5-fold cross-validation, DuAL-Net achieved an area under the receiver operating characteristic curve (AUC) of 0.698 (95% confidence interval: 0.659 to 0.737) for the top 100 ranked SNPs, substantially outperforming bottom-ranked SNPs (AUC = 0.479). Validation on an independent ADSP Alzheimer’s Disease Centers cohort (*n* = 5,570) confirmed generalizability, with top-ranked SNPs achieving AUC = 0.686 versus 0.516 for bottom-ranked SNPs. The framework successfully identified established risk variants, including rs429358 and rs7412, validating its ability to prioritize biologically relevant SNPs. DuAL-Net provides a generalizable approach for integrating local and global genomic information in Alzheimer’s disease risk prediction.

## Graphical Abstract

**Figure:**
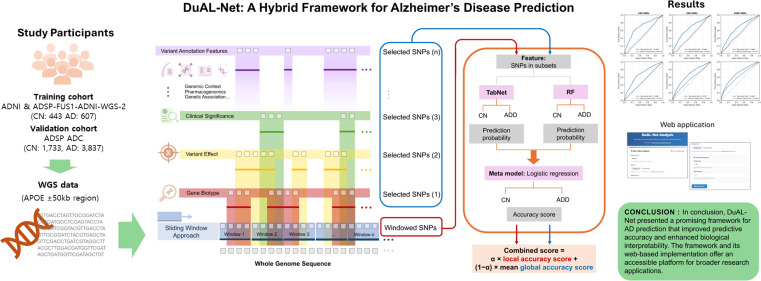

## Introduction

Alzheimer’s disease (AD) dementia, the most common type of dementia, is a progressive neurodegenerative disorder characterized by the accumulation of amyloid plaques and neurofibrillary tangles in the brain [1]. With the emergence of antiamyloid monoclonal antibody therapy for AD, early prediction of individuals at risk, especially before the onset of clinical symptoms, has become increasingly important, as these therapies are suggested to be more effective when initiated in the earlier stages of disease progression [2,3]. However, there are currently no effective screening methods, particularly before clinical and pathological changes manifest.

In this context, genomic data present a promising avenue for the early prediction of AD. Germline genomic data remain unchanged throughout an individual’s lifetime, enabling risk prediction from birth if accurate predictive models are developed. Additionally, twin studies estimate the genetic heritability of AD to be 50% to 70% [4,5], highlighting a strong genetic component and the potential of genomic data as a predictive tool. While common genetic variants collectively explain approximately 7% to 8% of AD heritability, the *APOE* region accounts for the largest portion [6]. Among known AD risk genes, the *APOE* ε4 genotype is the strongest genetic risk factor, increasing risk approximately 3-fold in heterozygotes and up to 15-fold in homozygotes [7].

Whole-genome sequencing (WGS) data offer more comprehensive information compared to other types of genomic data even though their high dimensionality poses a substantial challenge for analysis [8]. Moreover, the hierarchical structure of the genome presents an additional challenge, as both local interactions between nearby loci and long-range relationships between distant genomic regions can influence the endophenotype [9]. However, encoding or embedding methods that rely solely on raw WGS input often struggle to effectively capture these long-range relationships.

To address these limitations of WGS analysis, we proposed a hybrid framework that incorporates analyses of both local and long-range relationships. We reduced input dimensionality by dividing the WGS data into nonoverlapping local windows. In parallel, we annotated each single-nucleotide polymorphism (SNP) and reorganized the input based on these annotations, allowing the model to capture long-range relationships. This strategy possibly reflects the genome’s hierarchical structure and enables the model to improve disease prediction performance for complex disease such as AD.

Currently, most genome-based models yield only modest performance for predicting AD [10,11]. To address this challenge, our novel framework applied ensemble framework that combined 2 fundamentally different models: random forest (RF), a decision-tree-based method known for its robustness across various genomic applications [10–12], and TabNet, a deep learning model optimized for tabular data with built-in interpretability [13,14]. By integrating RF and TabNet through a stacking ensemble, the model leverages their complementary strengths, thereby enhancing overall performance.

In this study, we aim to (a) develop a novel hybrid framework optimized for WGS data analysis, (b) validate its potential for the prediction of AD dementia, and (c) identify novel SNPs that contribute substantially to AD dementia prediction within the context of the input WGS data.

The main contributions of this work are (a) a novel dual-network framework (DuAL-Net [Dual Approach Local-global Network]) that integrates local genomic patterns with global biological knowledge for SNP prioritization; (b) a demonstration that annotation-guided scoring improves the identification of disease-relevant variants, with rs429358 consistently ranked among the top SNPs; (c) empirical evidence that the integrated approach outperforms standard machine learning baselines; and (d) a generalizable framework that can be extended to other genomic regions and diseases where functional annotations are available.

## Methods

### Study participants

For the training cohort, this study included 1,566 participants drawn from 2 sources: the Alzheimer’s Disease Neuroimaging Initiative (ADNI; *N* = 809) [15] and ADNI-WGS-2 with Alzheimer’s Disease Sequencing Project (ADSP) Follow-Up Study (ADSP-FUS1-ADNI-WGS-2, *N* = 757) [16,17]. ADNI, launched in 2003 and extended through its phases (ADNI-1, ADNI-GO, ADNI-2, and ADNI-3), aims to discover biomarkers and monitor AD progression. This includes utilizing serial imaging studies, various biological markers, clinical and neuropsychological evaluations, and genomic information including WGS. The ADSP Follow-Up Study builds on the ADNI framework by extending longitudinal follow-up intervals and adding data for participants not previously sequenced, thus offering broader genomic coverage for AD-related investigations. From the combined pool, individuals diagnosed with mild cognitive impairment were excluded, yielding a final set of 443 cognitively normal (CN) participants and 607 with clinically confirmed AD dementia. Demographic information, *APOE* and whole-genome genotyping data, and clinical information were obtained from the ADNI data repository (https://adni.loni.usc.edu). All participants provided written informed consent, and the study protocol was approved by the Institutional Review Board at each data acquisition site.

We used the ADSP Alzheimer’s Disease Centers (ADC) cohort from ADSP Release 5 for validation. Standard ADSP quality control (QC) procedures were applied, and duplicate samples were excluded, yielding a total of 5,570 unique individuals, including 3,837 AD dementia and 1,733 CN participants. This validation cohort was fully independent of the ADNI training cohort. Table 1 presents the demographic characteristics of the training and validation cohorts.

**Table 1.** Demographic characteristics and *APOE* genotype distribution of participants. Values are presented as mean (SD) or number (%).

Characteristic	Training cohort ^a^		Validation cohort ^b^	
	CN (*n* = 443)	AD (*n* = 607)	CN (*n* = 1,733)	AD (*n* = 3,837)
Age, years (mean ± SD)	72.6 ± 6.3	74.0 ± 7.2	82.3 ± 5.7	68.1 ± 10.6
Female, *n* (%)	208 (47.0)	352 (58.0)	642 (37.0)	1,780 (46.4)
*APOE* genotype, *n* (%)				
ε2/ε2	2 (0.5)	1 (0.2)	8 (0.5)	3 (0.1)
ε2/ε3	57 (12.9)	19 (3.1)	162 (9.3)	74 (1.9)
ε2/ε4	7 (1.6)	14 (2.3)	18 (1.0)	88 (2.3)
ε3/ε3	257 (58.0)	194 (32.0)	1,048 (60.5)	1,239 (32.3)
ε3/ε4	110 (24.8)	278 (45.8)	454 (26.2)	1,850 (48.2)
ε4/ε4	10 (2.3)	101 (16.6)	35 (2.0)	526 (13.7)
ε4 carrier, *n* (%)	127 (28.7)	393 (64.7)	507 (29.3)	2,464 (64.2)

AD, Alzheimer’s disease; CN, cognitively normal; SD, standard deviation

^a^ The Alzheimer’s Disease Neuroimaging Initiative (ADNI) and ADNI-WGS-2 with Alzheimer’s Disease Sequencing Project (ADSP), which were used for model development.

^b^ ADSP Alzheimer’s Disease Centers (ADC) served as an independent replication cohort.

### WGS data

This study leveraged newly integrated WGS data (spanning the 50-kb region upstream and downstream of *APOE*) from both the ADNI and ADSP-FUS1-ADNI-WGS-2 cohorts. Sequencing was primarily conducted on Illumina platforms (HiSeq 2000, HiSeq X, or NovaSeq) with read lengths of either 100 or 150 bp. Library preparation was performed using standard Illumina reagents, and the resulting paired-end reads were aligned to the GRCh38 (hg38) human reference genome with BWA-MEM [18]. Picard tools were used to mark polymerase chain reaction duplicates and collect alignment metrics, followed by local realignment around insertions or deletions and base quality score recalibration with the Genome Analysis Toolkit (GATK) [19]. Joint variant calling across all samples proceeded via GATK HaplotypeCaller, adhering to best-practice guidelines, and the Genome Center for Alzheimer’s Disease implemented its variant calling pipeline (VCPA v1.0) to standardize QC measures [20,21]. Initial QC steps included verifying single-nucleotide variant concordance, identifying sex mismatches, detecting contamination, and evaluating relatedness (Pihat > 0.4). Additionally, sample-level QC excluded individuals with sex inconsistencies, call rates below 95%, or duplicated genetic profiles. At the SNP level, variants with a call rate below 95%, Hardy–Weinberg equilibrium *P* values <1 × 10^−6^, or a minor allele frequency <1% were removed. Further filters discarded genotypes with low genotype quality (GQ < 20) or insufficient read depth (DP < 10), and set to missing those variants exhibiting missing call rates >20%. Finally, any variants presenting as monomorphic or multiallelic were also excluded.

### Imputation

In our previous work, we systematically evaluated multiple imputation algorithms, including *k*-nearest neighbors (*k* = 1, 5, and 10), a generative adversarial network imputer, an iterative imputer, MissForest, and a simple imputer [22]. This evaluation involved artificially inflating the proportion of missing genotypes and subsequently comparing reconstructed data using accuracy, root mean square error, *R*-squared (*R*^2^), mean absolute error, and normalized root mean square error. Based on this evaluation, *k*-nearest neighbors imputation with *k* = 5 was selected as the optimal method. In the current study, we adopted the same pipeline to ensure methodological consistency with our previous approach.

After imputation, 50,000 bp upstream and downstream of the *APOE* region on chromosome 19 were extracted from the WGS data for analysis. This region included 14,094 SNPs.

### Genomic information of SNPs

**Fig. 1.**
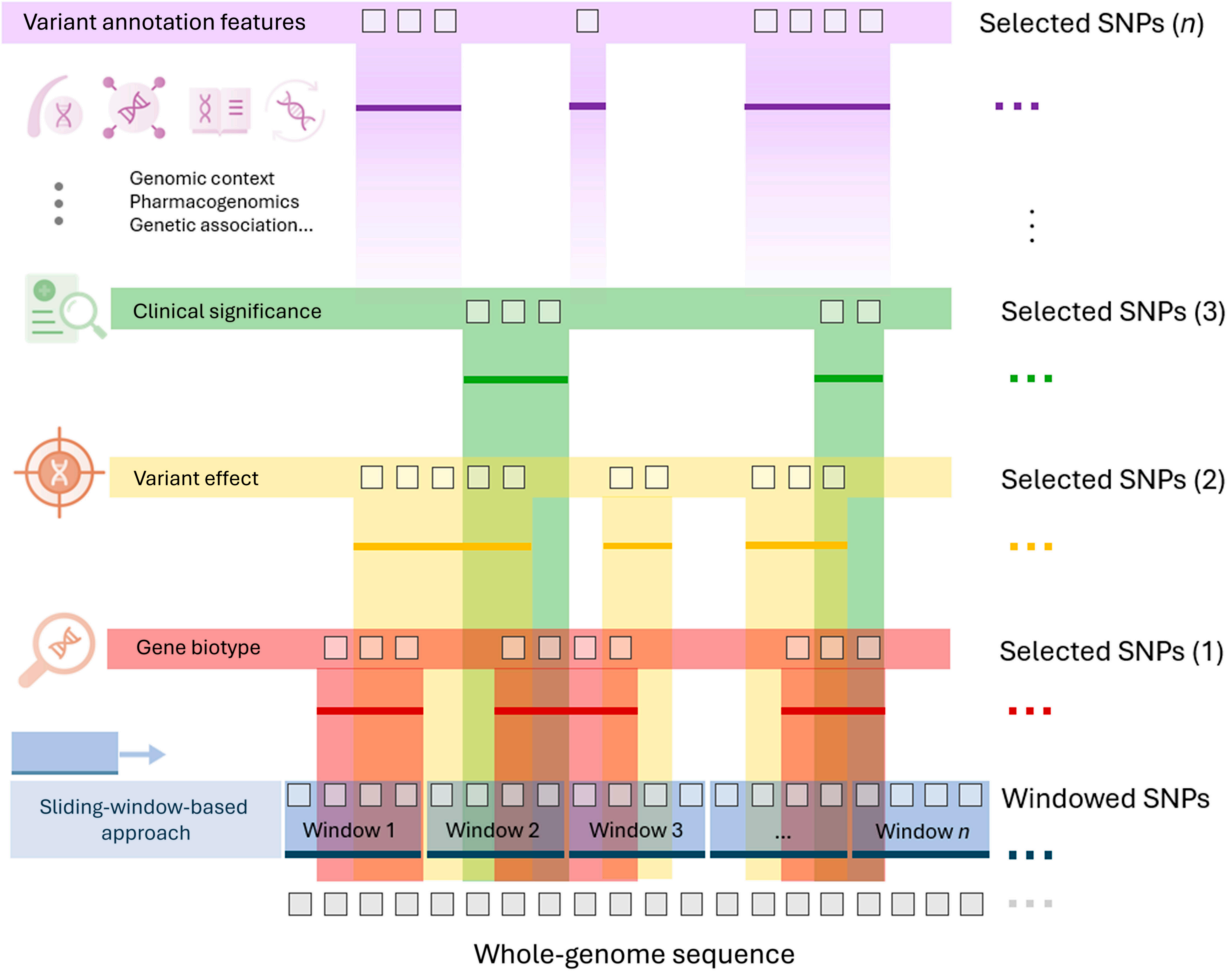
Annotation of genomic information to each SNP and grouping of SNPs by genomic windows or annotation categories. SNP, single-nucleotide polymorphism.

All retained SNPs were annotated using Ensembl genome annotation resources (Ensembl release 108, GRCh38). Figure 1 shows the annotation steps, which included determining the genomic context (exonic, intronic, and untranslated regions [UTRs]), regulatory elements (promoters and enhancers), predicted variant consequences, epigenetic features, and clinical significance categories. We leveraged the pyensembl library and Ensembl’s RESTful application programming interfaces (APIs) to systematically retrieve gene-, transcript-, and variant-level annotations.

After imputation, annotation was performed on the selected WGS data within a 50,000-bp region surrounding *APOE* using Ensembl’s RESTful APIs. Each SNP was divided into nonoverlapping 100-SNP windows and additionally grouped by annotation categories. The groups based on nonoverlapping windows and annotation were used as inputs for a model that combines TabNet and RF via out-of-fold (OOF) stacking.

### Model architecture

Our model architecture, which we named DuAL-Net, integrated TabNet [14] and RF to generate OOF predictions, which were subsequently used for metamodel training. The DuAL-Net framework was designed to leverage both local genomic context and global functional annotations to improve AD prediction accuracy. The complete implementation is available as open-source software on GitHub (https://github.com/taehojo/DuAL-Net) and accessible through a web server (https://www.jolab.ai/dualnet).

### Local probability modeling with OOF stacking

Local SNP interactions within genomic regions were captured through an OOF stacking ensemble strategy. After QC, individual SNP data were fragmented into nonoverlapping 100-SNP windows. The SNPs within each window were used as features for the classification model. Two different classifiers were trained to predict AD dementia (versus CN) using the SNPs within each window: a TabNet model, which employs a sequential attention mechanism designed for tabular data that selectively focuses on informative features, and an RF classifier, which is an ensemble of decision trees.

To estimate the predictive performance of each genomic window, the dataset was first partitioned into training and holdout subsets. Within the training subset, OOF predictions were generated for both TabNet and RF models using a 5-fold cross-validation (CV) framework. In each iteration, 4 folds were used for training the TabNet and RF models, with the remaining fold held out to obtain predictions.

The OOF predictions from both models were then used as features to train a logistic regression metamodel. The resulting stacked ensemble was subsequently evaluated on the holdout subset, and the classification accuracy obtained from this evaluation was assigned as the local performance score for all SNPs within the corresponding window. This score reflects the discriminative performance of SNPs in the window.

### Global annotation-based modeling

Annotation data from the previous “Genomic information of SNPs” section were converted into binary indicator variables. This encoding facilitated the selection of groups of SNPs sharing specific annotations. Specifically, we utilized the following annotation categories: genomic context (region none, indicating SNPs not located in exonic, intronic, or UTRs), gene biotype (to be experimentally confirmed [TEC], long noncoding RNA, protein coding, transcribed unprocessed pseudogene, and unknown), most severe consequences (3′ UTR variant, 5′ UTR variant, transcription factor binding site variant, unknown, intergenic variant, intron variant, missense variant, regulatory region variant, splice donor region variant, splice polypyrimidine tract variant, splice region variant, stop gained, and synonymous variant), and clinical significance (pathogenic, likely pathogenic, uncertain significance, drug response, other, risk factor, association, protective, and established risk allele).

For each annotation category, such as SNPs in protein-coding regions, SNPs with a missense consequence, or clinically pathogenic SNPs, a subset-specific classifier was trained using only SNPs belonging to that annotation group. Analogous to the local modeling strategy, the data were divided into training and holdout subsets, and within the training subset, TabNet and RF models were combined using an OOF stacking procedure implemented via 5-fold CV.

The stacked ensemble model was evaluated on the corresponding holdout subset to obtain a classification accuracy for each annotation-defined SNP group. This performance metric reflects the aggregate predictive information captured by SNPs sharing the same functional annotation. Each SNP then received a global accuracy score calculated by averaging the classification accuracies of all annotation groups in which it appeared.

### Combining local and global accuracy

The local and global scores were subsequently merged to produce a combined SNP prioritization metric. These scores reflect performance-based measures of discriminative ability derived from local genomic windows and global annotation-defined SNP groups. The combined score was computed using a weighting parameter *α* in the range [0,1], which determines the relative contribution of each score, according to the following formula:

$$\begin{array}{c} \text{Combined score}=\alpha \times \text{local accuracy score}\\ +\left(1-\alpha \right)\times \text{global accuracy score} \end{array}$$ (1)

An *α* of 1.0 used only the local model score, whereas an *α* of 0.0 used only the global score, and intermediate values provided a linear blend of the 2.

For the ensemble classifiers, RF was configured with n_estimators = 100, max_features = sqrt, and random_state = 42. TabNet was configured with feature dimension n_d = 8, attention dimension n_a = 8, n_steps = 3, learning rate = 0.001, batch size = 1,024, virtual batch size = 128, max epochs = 10, early stopping patience = 5, and mask type = entmax. All experiments used consistent random seeds for reproducibility.

To determine the optimal *α*, nested CV was employed. In the inner loop (5-fold CV within each outer training set), *α* values from 0.0 to 1.0 in increments of 0.1 were evaluated. For each candidate *α*, all SNPs were re-ranked according to their combined score, and the top 100 SNPs under that weighting were selected. Using these SNPs as features, the same RF–TabNet stacking ensemble with an OOF stacking strategy was trained and evaluated via inner CV. The *α* yielding the highest mean accuracy in the inner loop was selected as the optimal value for that outer fold.

The outer loop used 5-fold stratified CV for final performance evaluation. This nested design strictly separates hyperparameter tuning (inner loop) from performance evaluation (outer loop), preventing information leakage between model selection and final testing.

### Model evaluation and SNP prioritization

After selecting the optimal *α* via inner CV, all SNPs were assigned final combined scores and ranked from most to least likely to be associated with AD. To evaluate the methodology, we created multiple SNP panels of varying sizes (100, 500, and 1,000) from the top-ranked SNPs. For comparison, we also created corresponding panels of the same sizes using the bottom-ranked SNPs as a negative control.

Each SNP panel was evaluated using an ensemble classifier combining RF and TabNet through an OOF stacking strategy. Model performance was assessed on the holdout test set of each outer CV fold. Predicted probabilities for the test samples were generated using the trained ensemble model, and predictive performance was quantified using the area under the receiver operating characteristic (ROC) curve (AUC). Mean performance metrics and 95% confidence intervals (CIs) were estimated across outer CV folds.

### Validation on ADSP ADC

To assess the generalizability of DuAL-Net SNP rankings, we performed validation using the ADSP ADC cohort from ADSP R5 (3,837 AD dementia and 1,733 CN). This validation cohort is completely independent from the ADNI training cohort. SNP rankings derived from ADNI, based on the mean final scores across 5 outer CV folds, were transferred to the ADSP ADC dataset by genomic position. Using these transferred rankings, we constructed SNP subsets consisting of the top-ranked and bottom-ranked 100, 500, and 1,000 SNPs. Classification performance was evaluated within the ADSP ADC cohort using 5-fold stratified CV. In each fold, RF and TabNet models were trained to generate OOF predicted probabilities, which were subsequently combined using a logistic regression metamodel to obtain ensemble predictions. Predictive performance was quantified using AUC and accuracy. This transfer validation framework evaluates whether SNP prioritization learned from ADNI generalizes to predicting AD dementia status in an independent population.

### Baseline comparison

To contextualize DuAL-Net performance, 2 conventional machine learning baselines were evaluated using the same 5-fold stratified CV framework. First, logistic regression was trained on all 14,094 SNPs with standardized features (maximum iterations = 1,000). Second, univariate feature selection was performed using analysis of variance *F* test (SelectKBest) to select the top 100 SNPs within each training fold, followed by an RF classifier (n_estimators = 100) for classification. For both baselines, AUC values were computed for each fold, and statistical significance was assessed using paired *t* tests comparing fold-wise AUCs against DuAL-Net.

## Results

### Local SNP window analysis

We evaluated our DuAL-Net framework on 1,050 samples using an OOF stacking ensemble approach. The *APOE* region was divided into 140 contiguous windows of 100 SNPs each. The average classification accuracy across all windows was 0.612. Among the 140 windows evaluated, the lowest accuracy was 0.530 and the highest reached 0.762. A total of 56 windows achieved accuracies of 0.60 or higher. This approach identified genomic segments with above-average accuracy that were carried forward to the integrated analysis.

### Global annotation-based analysis

We performed a global analysis using functional annotations for each SNP. Analysis of individual annotation features revealed varying predictive power. SNPs with ClinVar clinical significance of “pathogenic” achieved the highest classification accuracy at 0.695. Variants labeled as “uncertain significance” or “drug response” yielded accuracies of 0.695 and 0.669, respectively. SNPs located in protein-coding genes predicted AD with 0.630 accuracy. The global analysis identified several annotation features with above-average predictive power for disease status.

### Integrating local and global scores (*α* optimization)

Through nested CV, the optimal integration weight *α* was determined to be 0.66 on average across folds. The integrated approach effectively prioritized disease-relevant variants, with rs429358 identified among the top-ranked variants in all 5 outer folds. The integrated DuAL-Net achieved an AUC of 0.698 (95% CI: 0.659 to 0.737) and an accuracy of 0.660 (95% CI: 0.617 to 0.703).

To directly compare the contributions of local and global components, we evaluated models using fixed weighting parameters under the same nested CV framework. The local-only model (*α* = 1.0) achieved an AUC of 0.682 (95% CI: 0.623 to 0.740), while the global-only model (*α* = 0.0) achieved an AUC of 0.652 (95% CI: 0.624 to 0.681). The integrated model (*α* = 0.66) significantly outperformed the global-only approach (paired *t* test, *P* = 0.010), confirming that annotation-based scoring provides complementary information to local genomic patterns. Table 2 presents a side-by-side comparison.

**Table 2.** Performance comparison of local-only, global-only, and integrated DuAL-Net models

Model	AUC	95% CI	Accuracy
Global-only	0.652	0.624–0.681	0.610
Local-only	0.682	0.623–0.740	0.653
Integrated	0.698	0.659–0.737	0.660

DuAL-Net, Dual Approach Local-global Network; AUC, area under the receiver operating characteristic curve; CI, confidence interval

### SNP ranking and ROC performance

Using the optimized combined score (*α* = 0.66), we ranked all SNPs from highest to lowest predicted relevance to AD. rs429358, the *APOE* ε4-defining variant, was identified among the top 100 ranked SNPs in all 5 folds, validating our method’s ability to identify established risk variants.

**Fig. 2.**
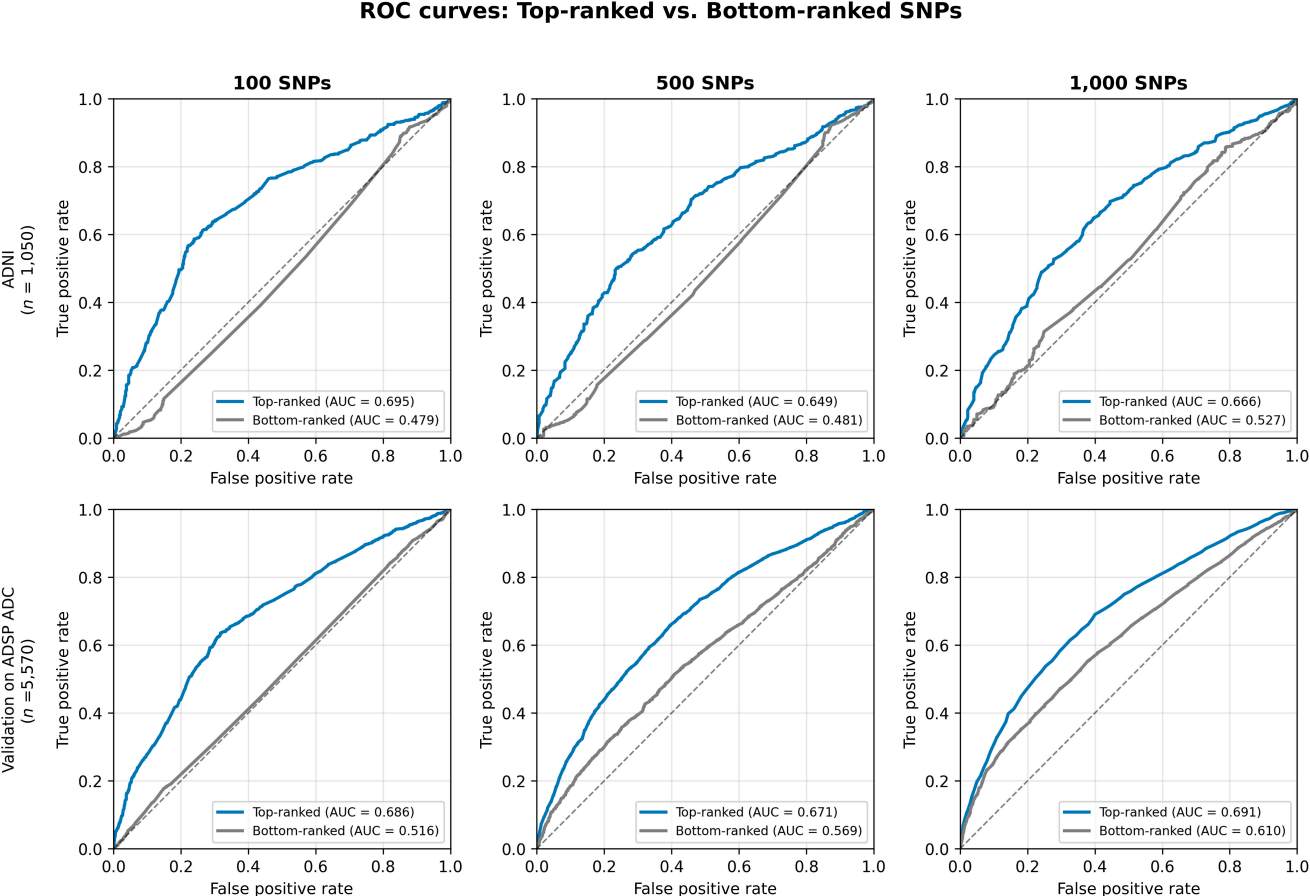
Receiver operating characteristic (ROC) curves comparing top-ranked and bottom-ranked SNP subsets across different sizes (100, 500, and 1,000 SNPs). Top row: ADNI cohort (*n* = 1,050). Bottom row: Validation on ADSP ADC cohort (*n* = 5,570). Top-ranked SNPs by DuAL-Net (blue) consistently showed higher AUC values than bottom-ranked SNPs (gray) in both cohorts, demonstrating the generalizability of the SNP rankings.

For the 100-SNP subset, the top-ranked SNPs achieved an AUC of 0.695, while bottom-ranked SNPs yielded an AUC of 0.479. For 500 SNPs, the top-ranked ones achieved 0.649 vs. 0.481 for the bottom-ranked ones. For 1,000 SNPs, the top-ranked ones achieved 0.666 vs. 0.527 for the bottom-ranked ones. Table 3 and Fig. 2 show the pattern that top-ranked SNPs consistently presented higher AUC values than bottom-ranked ones across other subset sizes.

**Table 3.** Classification performance of top-ranked versus bottom-ranked SNP subsets in the training and the validation cohort

Dataset	SNP subset	AUC (100)	AUC (500)	AUC (1,000)
Training cohort (*n* = 1,050)	Top-ranked	0.695	0.649	0.666
Bottom-ranked	0.479	0.481	0.527
Validation cohort (*n* = 5,570)	Top-ranked	0.686	0.671	0.691
Bottom-ranked	0.516	0.569	0.610

### Linkage disequilibrium analysis

To distinguish truly predictive SNPs from linkage disequilibrium (LD) proxies, we calculated pairwise LD (*r*^2^) between all variants and rs429358 (*APOE* ε4-defining variant) using ADNI genotype data and performed LD filtering. Using a commonly used threshold of *r*^2^ ≥ 0.2 for moderate LD, we identified 34 SNPs as potential LD proxies and excluded them from independent signal analysis. SNPs showing the highest LD with rs429358 included rs12721051 (*r*^2^ = 0.80), rs56131196 (*r*^2^ = 0.78), and rs157592 (*r*^2^ = 0.78). Notably, rs7412 (*APOE* ε2-defining variant) showed a very low LD with rs429358 (*r*^2^ = 0.035), confirming that these 2 *APOE*-defining variants represent independent genetic signals. After LD filtering, the remaining top-ranked SNPs represent independent signals beyond primary *APOE* ε4 effect.

### Validation on ADSP ADC

To assess generalizability, we performed validation using the ADSP ADC cohort from ADSP R5 (*n* = 5,570; 3,837 AD dementia and 1,733 CN), which is completely independent from the ADNI training cohort. We applied the SNP rankings derived from ADNI to this external dataset. Top-ranked SNPs by DuAL-Net consistently outperformed bottom-ranked SNPs across all subset sizes: for 100 SNPs, top-ranked achieved AUC = 0.686 vs. bottom-ranked AUC = 0.516 (gap = +0.170); for 500 SNPs, top-ranked achieved AUC = 0.671 vs. bottom-ranked AUC = 0.569 (gap = +0.102); and for 1,000 SNPs, top-ranked achieved AUC = 0.691 vs. bottom-ranked AUC = 0.610 (gap = +0.081). These results, summarized in Table 3, demonstrate that DuAL-Net identifies genuinely predictive variants that generalize to independent cohorts.

## Discussion

This study proposes DuAL-Net as a proof-of-concept framework for integrating local genomic patterns with global biological annotations in the *APOE* region. Our primary contribution is methodological: demonstrating that annotation-guided scoring substantially improves variant prioritization compared to using genomic data alone.

DuAL-Net demonstrates that integrating complementary sources of genomic information improves predictive performance. The local-only model (*α* = 1.0) achieved an AUC of 0.682, the global-only model (*α* = 0.0) achieved an AUC of 0.652, and the integrated model with optimized weighting (*α* = 0.66) achieved an AUC of 0.698. The integrated model significantly outperformed the global-only approach (paired *t* test, *P* = 0.010). This improvement indicates that combining both analyses effectively captures the multilayered structure of WGS data and provides additional information for AD prediction. By using external annotations to guide SNP selection, we created a biologically informed framework that reduces the WGS feature space and employs a hierarchical approach, enhancing both interpretability and computational efficiency. Furthermore, our framework provides a generalizable strategy for incorporating diverse multimodal data into WGS analysis. Importantly, the validation on the ADSP ADC cohort (*n* = 5,570) demonstrated that ADNI-trained SNP rankings generalize to independent data, with top-ranked SNPs consistently outperforming bottom-ranked variants (AUC: 0.686 vs. 0.516). This performance reflects the inherent limitations of genetic-only prediction from a single genomic region.

Our ROC analyses across different subset sizes (100, 500, and 1,000 SNPs) consistently showed that top-ranked SNPs carried substantially more predictive power than bottom-ranked variants. The top 100 SNPs achieved an AUC of 0.695, while bottom-ranked SNPs performed near chance level (AUC = 0.479). This pattern held across all subset sizes, with the top 500 SNPs achieving 0.649 and the top 1,000 SNPs achieving 0.666. These findings indicate that DuAL-Net effectively identifies SNPs predictive of AD. Therefore, DuAL-Net offers a supervised feature selection approach that prioritizes features based on their relevance to the prediction task.

To assess the contribution of rs429358 to the observed performance differences between top-ranked and bottom-ranked SNP subsets, we performed an ablation analysis in which rs429358 and its LD proxies (34 SNPs with *r*^2^ ≥ 0.2) were removed from the top 100 ranked SNPs. In the ADNI cohort, the ablated top-ranked set (without *APOE* ε4-associated variants) achieved an AUC of 0.661, compared to 0.697 with the full top 100 and 0.477 for the bottom 100. In the ADSP ADC validation cohort, the ablation effect was minimal (AUC: 0.680 vs. 0.686), while the bottom-ranked set achieved only 0.516. These results indicate that while rs429358 is a major contributor to classification performance, the remaining top-ranked SNPs retain substantially more predictive power than bottom-ranked variants (gap: +0.184 in ADNI and +0.164 in ADSP ADC after ablation). The identification of rs429358 as a top-ranked SNP across all 5 CV folds serves as a validation of DuAL-Net’s ability to correctly prioritize established risk variants from among 14,094 candidates. In future genome-wide applications, the relative contribution of any single variant would be more distributed across multiple risk loci.

This study has several limitations that should be acknowledged. First, our analysis focused exclusively on the *APOE* region (±50 kb), and extension to other established AD risk loci (*BIN1*, *CLU*, *TREM2*, *ABCA7*, *CR1*, and *PICALM*) represents an important direction for future work. The DuAL-Net framework is designed to be generalizable to other genomic regions where functional annotations are available. Second, this study did not compare against polygenic risk scoring methods (PRS-CS and LDpred2) that aggregate effects across the genome; such comparison would require genome-wide data beyond the *APOE* region analyzed here. Third, the predictive performance achieved in this study (AUC = 0.698) remains insufficient for direct clinical application as an independent risk prediction tool. Nevertheless, our findings highlight that leveraging functional annotations in conjunction with modeling based on genomic data leads to more effective variant prioritization than approaches that rely exclusively on genomic sequence information. Fourth, while we validated on the ADSP ADC cohort (*n* = 5,570), further validation on diverse ancestral populations is needed to assess broader generalizability.

In conclusion, DuAL-Net presents a novel framework for AD risk prediction that integrates local window analysis with global annotation-based modeling. The top-ranked SNPs identified by our framework demonstrated substantially higher predictive power than bottom-ranked variants, validating our approach for SNP prioritization. This framework provides a foundation for future genomic risk prediction studies. The framework achieved an AUC of 0.698 (95% CI: 0.659 to 0.737), significantly outperforming conventional baselines (logistic regression *P* = 0.019, univariate selection *P* = 0.026) while consistently identifying rs429358 across all CV folds. The top-ranked SNPs identified by our framework demonstrated substantially higher predictive power than bottom-ranked variants, validating our approach for SNP prioritization. This work provides a foundation for extension to genome-wide studies and multimodal risk prediction incorporating clinical and imaging biomarkers.

## Data Availability

The whole-genome sequencing data used in this study were obtained from the ADNI database and the ADSP Follow-Up Study. Access to these datasets requires approval from the respective data access committees. The ADNI data are available to qualified researchers upon registration and approval at https://adni.loni.usc.edu. The code for DuAL-Net is publicly available at https://github.com/taehojo/DuAL-Net, and the web-based implementation can be accessed at https://www.jolab.ai/dualnet.
